# Food Choices after Cognitive Load: An Affective Computing Approach

**DOI:** 10.3390/s23146597

**Published:** 2023-07-21

**Authors:** Arpita Mallikarjuna Kappattanavar, Pascal Hecker, Sidratul Moontaha, Nico Steckhan, Bert Arnrich

**Affiliations:** 1 Digital Health—Connected Healthcare, Hasso Plattner Institute, University of Potsdam, 14482 Potsdam, Germanybert.arnrich@hpi.de (B.A.); 2 Institute for Social Medicine, Epidemiology and Health Economics, Charité, 10117 Berlin, Germany

**Keywords:** cognitive load, eating behaviour, machine learning, physiological signals, photoplethysmography, electrodermal activity, sensors

## Abstract

Psychology and nutritional science research has highlighted the impact of negative emotions and cognitive load on calorie consumption behaviour using subjective questionnaires. Isolated studies in other domains objectively assess cognitive load without considering its effects on eating behaviour. This study aims to explore the potential for developing an integrated eating behaviour assistant system that incorporates cognitive load factors. Two experimental sessions were conducted using custom-developed experimentation software to induce different stimuli. During these sessions, we collected 30 h of physiological, food consumption, and affective states questionnaires data to automatically detect cognitive load and analyse its effect on food choice. Utilising grid search optimisation and leave-one-subject-out cross-validation, a support vector machine model achieved a mean classification accuracy of 85.12% for the two cognitive load tasks using eight relevant features. Statistical analysis was performed on calorie consumption and questionnaire data. Furthermore, 75% of the subjects with higher negative affect significantly increased consumption of specific foods after high-cognitive-load tasks. These findings offer insights into the intricate relationship between cognitive load, affective states, and food choice, paving the way for an eating behaviour assistant system to manage food choices during cognitive load. Future research should enhance system capabilities and explore real-world applications.

## 1. Introduction

Elevated Body Mass Index (BMI) due to overweight and obesity is a risk factor for cardiovascular disease, stroke, diabetes, musculoskeletal disorders, and some cancers [1]. Statistics from the World Health Organisation in 2016 show that more than 1.9 billion adults over 18 were overweight, and 650 million adults were obese [1]. Dietary habits and a sedentary lifestyle are the main causes of overweight and obesity [1]. Some factors identified for excessive food intake leading to obesity are socioeconomic conditions, educational level, stress, and negative emotions [2,3,4]. Understanding how cognitive load affects eating behaviours and other weight-related behaviours could improve the effectiveness of obesity prevention and intervention programs [5].

Cognitive load is a multidimensional construct that represents the load imposed on an individual’s cognitive system to perform or accomplish a task [6]. Cognitive load can be measured using a variety of subjective, performance, behavioural, and physiological measures [7,8]. Subjective measures of cognitive load are based on self-reports. They may use rating scales or questionnaires to assess the subjects’ perceived level of mental effort [7,9]. Performance measures are expressed by task completion time, speed, correctness, critical errors, and false starts [7,9,10]. Behavioural measures of cognitive load focus on observing patterns of interactive behaviour, such as linguistic or dialog patterns, and even text input and mouse click events [7,11,12,13]. Physiological measures of cognitive load focus on the body’s physical responses during task performance. These measures include brain waves, eye activity, heart rate, and skin conductance response [7,14,15,16].

Recently, the objective classification of cognitive load has been conducted through the measurement of physiological signals both in laboratory and real-world settings [8,17,18]. However, these studies have yet to specifically examine the effects of cognitive load on eating behaviour. In the fields of psychology and nutritional science, researchers have induced stress and cognitive load in laboratory conditions and assessed their impact using self-reported questionnaires, focusing on various eating outcomes, such as the consumption of unhealthy snacks and high-calorie foods [19,20]. It is worth noting that these studies, which investigate the relationship between cognitive load and eating behaviours in laboratory settings, rely on subjective measures, highlighting the need for validating cognitive load outcomes with objective measures. Furthermore, consumer and eating behaviour researchers stress the importance of employing objective and non-obtrusive measures to gain insights into subconscious behaviours [21,22,23]. Although recent research in food recommendation systems has explored context-based approaches involving factors like heart rate, location, and glucose level [24], the specific aspect of cognitive load leading to higher calorie intake has yet to be objectively addressed.

In the scope of the EATMAPS project, this study aims to address the research gap by objectively classifying cognitive load and examining its impact on calorie consumption. By addressing gaps in the current research landscape, this research contributes to a deeper understanding of the relationship between cognitive load and eating behaviour. The main goal is to develop an eating behaviour assistance system based on a person’s cognitive load. To develop such a system, we collaborated with the company Oviva AG. They have developed a mobile application to monitor the eating habits of diabetic patients based on the images they take of the food they eat. Oviva AG performs image classification of foods into 18 categories. However, objective data on affective states or cognitive load are needed to determine if these affect the patient’s eating behaviour. This study’s purpose is to establish a robust framework for data collection within a controlled experimental environment, enabling comprehensive data acquisition. This framework will not only facilitate future data collection efforts but also be instrumental in training models for the development of an eating behaviour assistant system, empowering individuals to adopt and maintain healthy eating behaviours. The aforementioned study also forms the basis for analysing the extensive data collected in everyday life within the EATMAPS project, encompassing physiological signals, global positioning system data, affective states questionnaires, and food images, amounting to approximately 750 h of data [25]. In this study, we are addressing three research questions:

- How can a robust framework be developed to induce cognitive load and capture cognitive load data utilising subjective and objective modalities while also capturing information about food consumption?
- Can a generalised classification model be developed to effectively differentiate between High Cognitive Load (HL) and Low Cognitive Load (LL) levels with high accuracy while mitigating overfitting?
- Is there a relationship between cognitive load, affective states, and food choice?

Our contributions in this study can be summarised as follows:

- We developed a data collection protocol to distinguish between HL and LL and included eating activity in the study. To avoid bias due to interruptions by the experimenter in giving instructions and presenting the tasks, we developed an experimentation software using PsychoPy [26] version 2021.1.4, a cross-platform Python package.
- This paper presents a valuable resource consisting of multimodal data collected from 12 subjects for approximately 2.5 h per subject, encompassing HL and LL tasks. Researchers can request access to this dataset, and this is the first dataset to provide simultaneous availability of calorie consumption data and physiological signals related to cognitive load.
- The study employed feature selection techniques to identify eight relevant features from the extensive set of features extracted from the Shimmer GSR+ device sensor data. By reducing the complexity of the machine learning model, these selected features contribute to improving the efficiency of cognitive load classification. Additionally, we implemented a comprehensive evaluation approach, incorporating nested cross-validation hyperparameter optimisation in our classification pipeline with Leave-One-Subject-Out (LOSO). This methodology assesses the generalisation performance of a model across different subjects [27]. In LOSO cross-validation, the data of one subject is held out as a test set, and the model is trained on the remaining data from the other subjects. This process is repeated for each subject, such that each subject’s data is used once as the test set while the model is trained on the remaining subject’s data.
- The research findings highlight an intriguing relationship between cognitive load, negative affect, and food choices. Specifically, the study reveals that 75% of the subjects with higher negative affect after HL tasks reported increased consumption of specific foods. This discovery offers valuable insights into the complex interplay between cognitive load, affective states, and eating behaviours, potentially contributing to developing an eating behaviour assistant system to promote healthier eating habits in individuals facing cognitive demands and negative emotional states.

**Figure 1:**
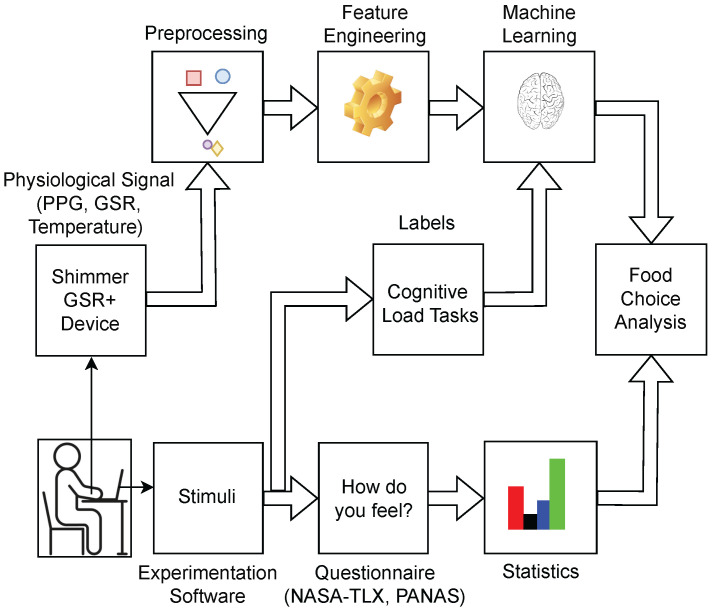
Overview of the experimental framework, with the three types of data collected (physiological signals, food calories, and questionnaires) and the data processing steps to find and analyse trends in eating behaviour after cognitive loads. Abbreviations: GSR—Galvanic Skin Response, PPG—Photoplethysmography.

The experimental framework overview is presented in Figure 1, illustrating the data collection process involving physiological signals from Shimmer GSR+ device, questionnaires, and labels. The collected physiological signals undergo preprocessing, feature extraction, and classification using cognitive load task labels extracted from experimentation software. The questionnaires obtained from experimentation software are also subjected to statistical analysis. Subsequently, the classified cognitive load data, statistically analysed questionnaires, and food consumption records are integrated for food choice analysis. The study encompasses multiple stages, including data collection, preprocessing, feature extraction, classification, statistical analysis of questionnaires, and food choice analysis.

## 2. Related Work

This section explores various aspects related to the investigation of cognitive load, including sensor types, experimental setup, subject population, algorithm cross-validation, and feature selection used in the literature for cognitive load classification. Additionally, we review studies that have made cognitive load data available to other researchers. Furthermore, we delve into studies examining the relationship between eating behaviour and cognitive load.

### 2.1. Cognitive Load and Physiological Sensors

Various studies have employed physiological sensors, such as Electrocardiogram (ECG), Photoplethysmography (PPG), Galvanic Skin Response (GSR), Electroencephalogram (EEG), and Electrooculography (EOG) to measure cognitive load [8]. Some studies have focused on using a single type of sensor (unimodal sensors), while others have utilised combinations of sensors (multimodal sensors) to classify cognitive load.

For example, Gasparini et al. [28] collected PPG sensor data from 16 adult participants, extracting 20 significant Convolutional Neural Network (CNN) features using the relief feature selection method. They achieved a binary classification accuracy of 79% using hold-out cross-validation. However, it is important to note that the lack of transparency regarding the specific features used in the classification process is a limitation that should be considered. Saha et al. [29] gathered EEG data from four subjects and achieved an accuracy of 86.33% using Monte Carlo cross-validation. However, the small dataset and absence of LOSO cross-validation raise concerns about the study’s generalisability and model performance on new subjects.

Several studies have investigated the use of multiple sensors in different contexts. Nourbakhsh et al. [30] utilised GSR and eye blinking data from 13 subjects. They achieved a classification accuracy of 75% by employing LOSO cross-validation. In another study, Fan et al. [14] collected ECG and EEG data from 20 subjects in a 28 min experimental setup. They employed Support Vector Machine (SVM) with 5 features (reduced from 14 features using principal component analysis) and achieved an accuracy of 80% in classifying mental load. The dataset was divided into 75% for training and 25% for testing purposes. Similarly, Barua et al. [15] conducted a study involving 66 participants who performed a simulated driving task. They employed a binary classification task using a Random Forest (RF) algorithm with 42 features. The achieved classification accuracies were 74% and 88%. The dataset was split into a training set comprising 70% of the data and a test set containing 30%. It is worth noting that neither Fan et al. [14] nor Barua et al. [15] employed LOSO cross-validation. Integrating LOSO cross-validation would have provided a more robust evaluation of the model’s performance and generalisability to new subjects.

Several recent studies have contributed to the field of cognitive load research by making their datasets available to other researchers. For instance, Markova et al. [31], Gjoreski et al. [16], Beh et al. [32], and Oppelt et al. [33] have released datasets for cognitive load analysis. However, it is important to note that while these datasets provide valuable resources for researchers, none of them have published the physiological signals related to cognitive load and food consumption.

### 2.2. Cognitive Load and Eating Behaviour

Previous research has primarily focused on measuring cognitive load objectively for various target groups, such as drivers and students, to improve safety and comprehension [15,34]. However, there is a scarcity of studies objectively investigating the specific effects of cognitive load on eating behaviour. Research in psychology and nutrition science has identified subjective associations between measured cognitive load levels and various eating outcomes, including an increased tendency to consume unhealthy snacks [19,35].

**Figure 2:**
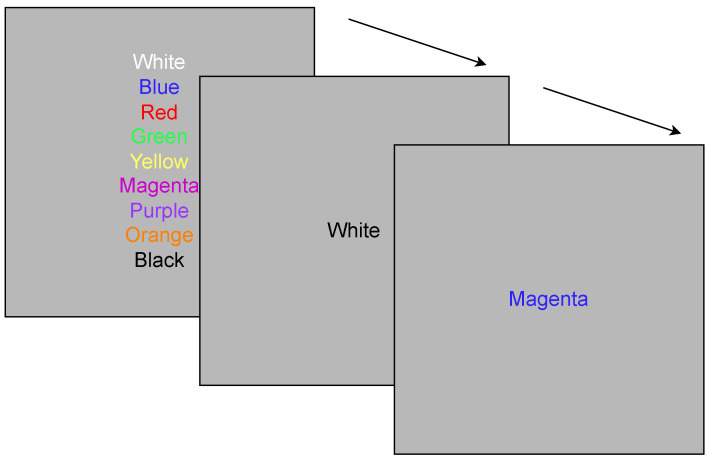
Demonstration of the Stroop task: In the first block, the font colours are identical to the colour name; in the second and third blocks, the Stroop task is demonstrated with each colour written in a different font colour.

Lattimore et al. conducted a study with female restrained eaters (who restrict their food intake to prevent weight gain or promote weight loss) and unrestrained eaters [36]. These subjects were asked to play four different types of Stroop tasks. In this task, the subjects were asked to say the colour of the string, not what the string said; the game is as demonstrated in Figure 2. In this study, restrained eaters ate significantly more than unrestrained eaters after Stroop tasks with ego threat (identifying the colour each string was printed in as quickly as possible using the red, green, blue, or yellow buttons on a response pad). Ward et al. conducted two experiments to examine the effects of HL on restrained and unrestrained eaters [20]. The first experiment found that restrained eaters ate more during HL tasks. In the second experiment, it was observed that subjects ate more during tasks that engaged their attention than during tasks that did not engage attention. Zimmerman et al. conducted a study investigating the impact of advertisement and cognitive load on food choices [35]. The study involved four groups with varying levels of cognitive load and food advertisements. After completing their respective tasks, the participants were offered snacks. The results indicated that participants in the HL group who were exposed to food advertisements consumed significantly more unhealthy snacks and calories than those in the other groups.

However, none of the three authors mentioned above objectively estimated cognitive load or tested the effects of different levels of cognitive loads on the same subject to examine within-subject effects. In this study, we aim to overcome these limitations and pave the way for the development of an eating behaviour assistance system.

## 3. Materials and Methods

This section describes the methods used to develop a experimental sessions representing the first step in developing an eating behaviour assistance system. Here we present the details of the design and implementation of the data collection, the use of the experimentation software to perform different tasks, the calorie calculation, the labelling of the tasks performed by the subjects, a pipeline to classify HL and LL tasks, and finally aggregate all the data to visualise the effect of cognitive load tasks on food choice.

### 3.1. Data Collection Setup

Before data collection, we obtained approval for the study from the ethics committee of the University of Potsdam, which was granted in March 2021. We then recruited subjects by advertising the study on the university campus and in student dormitories. Interested subjects contacted us by email. Subjects interested in the study were asked to fill out a form with their demographic information and answer health-related questions. Based on their answers, healthy subjects without diabetes, hypertension, or mental disorders were recruited. Participating subjects were sent an information sheet to brief them on the data collection. Subjects signed the informed consent form before the start of the study. On the day of their participation, we asked subjects to fast for 3 h before the study and not to consume food or coffee. We also informed them that they would be offered snacks after the experiment as they would be fasting for more than 4 h at the end of the experiment.

We recruited 14 subjects for the study. However, data from only 12 subjects were analysed, as data collection from two subjects failed due to technical issues with sensors. The 12 subjects averaged 27.9±2.7 years of age, 173.16±10.6 cm in height, and 70.29±11.6 kg in weight and were from either Asia or Europe.

Subjects were asked to wear the Shimmer3 GSR+ (Shimmer Research Ltd., Dublin, Ireland) device. This device in our study was used to collect PPG, GSR, accelerometer, and temperature sensor data from the non-dominant hand with a sampling rate of 51.2 Hz. PPG sensors measure heart activity. GSR measures the electrical property of the skin manifested in sweat due to the Sudomotor Nerve Activity (SMNA). At the beginning of the experiment, subjects were asked to shake their non-dominant hand three times to synchronise the different sensor data streams with the experimentation software, which was used to display stimuli for different tasks.

### 3.2. Experimentation Software for Lab Task

**Figure 3:**
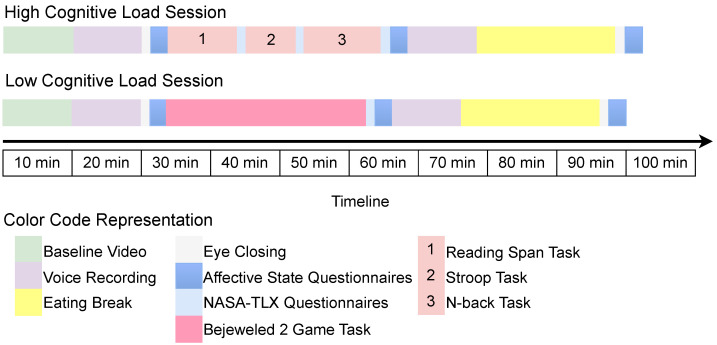
High and low cognitive load recording session sequence (**left** to **right**).

To conduct the experimental sessions, we developed experimentation software using PsychoPy version 2021.1.4, a cross-platform Python package [26]. We used the experimentation software to develop the sequences of cognitive tasks, stimuli, questionnaires, and instructions for the study and present them to subjects without interruption from the experimenter. We scheduled two data collection experimental sessions in a laboratory setting on two consecutive days to induce HL and LL tasks. In the first experimental session, subjects were randomly assigned to HL or LL sessions to avoid eating bias. The two experimental sessions included baseline video, voice recordings, cognitive load tasks, and questionnaires implemented in the software version 1.1.0. The sequence of tasks to be performed by the subject was displayed by the software, and the sequence is shown in Figure 3.

In the beginning, there was a ten-minute baseline video to relax the subjects in both cognitive sessions. Then, subjects had to record various speech prompts designed to elicit affective states. Subsequently, subjects had to close their eyes for one minute before answering affective states questionnaires (Positive and Negative Affect Schedule (PANAS)). The closing of the eyes served as a baseline for the acquisition of the EEG data. However, EEG data analysis is beyond the scope of this paper.

After answering the questionnaire, subjects started the cognitive load tasks. In the HL session, subjects performed three tasks that induced HL. At first, they performed a Reading Span task [37] at their own pace, which took approximately 7 to 10 min. In this task, subjects were asked to read logical and illogical sentences and to press the right arrow key if the sentence was logical or the left arrow key if the sentence was illogical on the keyboard [37,38]. In addition, subjects were instructed to memorise the numbers displayed between readings and enter them in the display of the software after the round. Subjects performed three sets of this Reading Span task.

The second task was the Stroop task, which took approximately 5–8 min. Subjects had three types of Stroop tasks. The first type of Stroop task was a self-paced task in which subjects read the colour name, printed it in mismatched colours, and pressed the space bar to advance to the next word [39]. Figure 2 demonstrates the Stroop task. In the second Stroop task, subjects had to read the colour name printed in mismatched colours with time pressure [38]. In the third Stroop task, in addition to displaying the colour name, they were also presented with high- and low-pitched tones, and they were asked to count the high-pitched tones and enter them at the end of the round [38].

**Figure 4:**
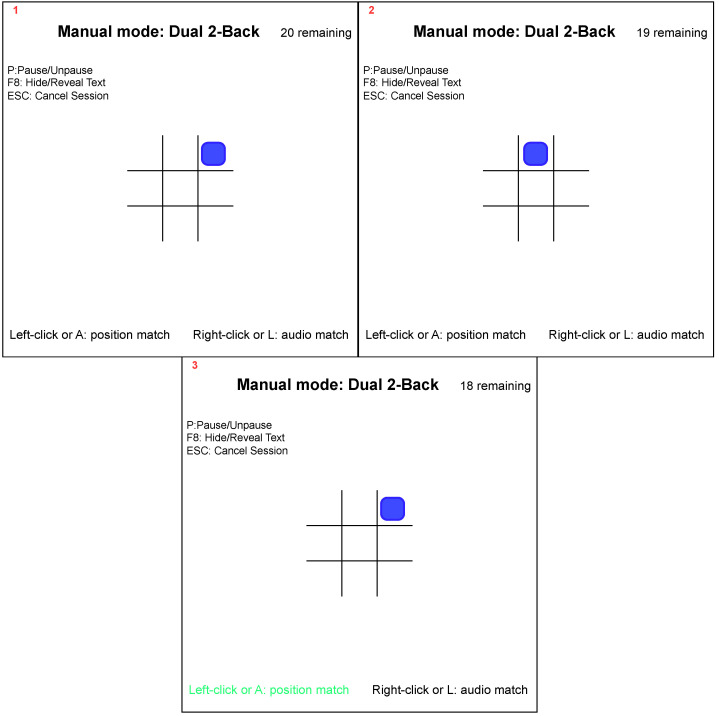
High-cognitive-load 2-back task demonstration. The blue square which appeared in the first block repeats its position in the third block.

The final task, with a HL, was the dual-N-back task. Here, subjects had to simultaneously recall a sequence of spoken letters and positions of a square and recognise whether the letter or position of the square matched the one that had previously appeared [40]. If the subject’s performance in the first level of 1-back task (in which the subject had to recall only the square and letter displayed or announced before the current square or letter) was more than 80% correct, the identification length difficulty level was increased to 2-back, 3-back, and the like. Figure 4 demonstrates the 2-back task. The numbers written in red on the top left in the Figure 4 represent the flow of explanation of the block. In the first block, we have the blue square in the third position, first row. The blue square is present in the last row in the second block. In the third block, that is, after 2-back, the square position repeats its position. Since this position matches the square’s position in the first block, the subject would be required to press the left mouse button during the third block to indicate the correct occurrence of 2-back. The N-back task was played for only about 10 min. The N-back task was implemented by opening the Brain Workshop software version 4.8.4 [41].

After each of the three tasks during HL session, subjects had to answer the National Aeronautics and Space Administration Task Load Index (NASA-TLX) questionnaire. NASA-TLX is a six dimensional subjective assessment tool to measure workload [42,43,44]. After the final NASA-TLX questionnaire, subjects again answered the affective states questionnaires and the recorded voice prompts about affective states, followed by a 20-min break to eat. Finally, subjects were asked to close their eyes for one minute and then open them again to answer further affective states questionnaires.

During the LL session, subjects were engaged in tasks similar to the HL session, except for excluding three cognitive tasks, namely Reading Span, Stroop task, and N-back task. Instead, they were given the task to play Bejeweled 2, a computer game involving matching and sequencing jewel shapes [45]. This game is considered a relaxing and LL task and was played for approximately 25 min. The game aims to combine at least three similar jewel shapes to score points. The game was accessed on the experimentation software via Steam Software (version 2021.05.18, Valve Corporation, Bellevue, WA, USA).

### 3.3. Labels and Analysis

Timestamps generated by the experimentation software for each task were used to label the data. We also calculated the NASA-TLX scores for the HL and LL sessions, as mentioned in the work of Hart et al. [42,43] and Rubio et al. [44]. NASA-TLX calculates the workload for each task. They were used to analyse the classification results. Also, we calculated the PANAS scores as mentioned in the work of Watson et al. [46] for positive and negative affective states. Positive affect reflects emotions such as interest, excitement, strength, enthusiasm, pride, alertness, inspiration, determination, attention, and being active. In contrast, negative affect reflects negative emotions such as stress, upset, guilt, fear, hostility, irritability, shame, nervousness, and jitter. PANAS scores can range from 10 to 50. Lower scores indicate lower levels of positive/negative affect, and higher scores indicate higher levels of positive/negative affect.

We performed a Shapiro–Wilk test to examine the normality of the scores of PANAS and NASA-TLX collected in both sessions. Then, we performed the *t*-test and Friedman statistical test to determine the significant difference between and within groups based on the Shapiro–Wilk test results.

### 3.4. Calorie Calculation and Food Choice Analysis

**Figure 5:**
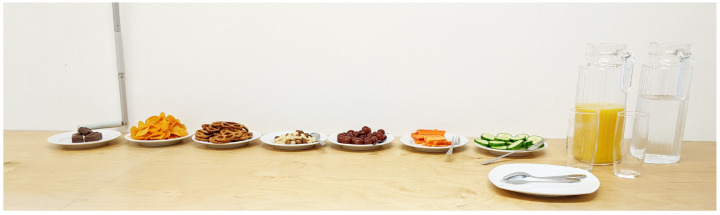
Arrangement of snacks: (**left** to **right**) 3 chocolate bars, potato chips, salted pretzels, nuts, black grapes, carrot slices, cucumber slices, orange juice, and water.

During the study, subjects were given a 20 min break and were offered following snacks: 3 chocolate bars without wrappers (3 × 18 g), potato chips (50–60 g), pretzels (70–80 g), nuts (50–60 g), grapes (70–85 g), carrot slices (70–85 g), cucumber slices (70–85 g), water (500 mL), and orange juice (500 mL). The foods were arranged as shown in Figure 5. We measured the weight of the snacks before and after consumption to calculate the calories consumed based on the calorie information on the food packaging. For the products for which calorie information was not available, we used the nutritionix website [47].

The Shapiro–Wilk test was performed on particular subjects grouped based on the results of the Friedman statistical test on PANAS to verify the distribution of calories and amount of each food consumed. We performed either a *t*-test or a Wilcoxon signed-rank test based on the data distribution to determine the significant difference in food consumption between the two sessions.

### 3.5. Cognitive Load Classification

In this section, we explain the pipeline to perform binary classification of the cognitive load states of the subjects.

**Synchronisation and Segmentation:** First, we synchronised the Shimmer3 GSR+ data with the software labels output using the magnitude of acceleration data. The magnitude of the acceleration was high at the beginning of the experiment due to hand tapping. From this point, we obtain the timestamp of the labels generated by PsychoPy. We then segmented the data into baseline, questionnaires, voice, eating, and cognitive load.

**Data Preprocessing:** To remove the gravitational component and the high frequency components of the accelerometer data, we applied a Butterworth bandpass filter with a filter order of 4 to the signal between 0.5 Hz and 6 Hz. Then, the magnitude of the accelerometer was calculated from the 3-axis.

To calculate heart rate from the PPG signal, the raw PPG signal was Butterworth-bandpass-filtered between 0.5 and 8.0 Hz with an order of 4 using the Neurokit2 Python package [48]. Then, the peaks in the PPG signals were identified by finding the local maxima by comparing the adjacent values. In this study, we identified the peaks using the SciPy Python package. We then calculated the heart rate by checking the distance between each peak [49]. If the distance between the corresponding peak was greater or less than 10% of the previous distance, these peaks were not considered heartbeats.

To derive features from raw GSR, we separate the tonic and phasic components using the CVXEDA algorithm as these components have different time scales, and relationships to the triggering stimuli [50]. Tonic components represent slow drifts of the baseline and are fluctuations in skin conductance [50]. The phasic component reflects the short-term response to a stimulus [50]. These components identify features that can discriminate between cognitive load states. In addition, using the same algorithm, we also derived SMNA, whose features are also used to discriminate between load levels. This algorithm is based on Bayesian statistics, convex mathematical optimisation, and sparsity [50].

**Windowing and Artefact Removal:** The preprocessed signals were segmented into 2 min windows with 75% overlap. We chose 2 min windows because preliminary testing showed that shorter window lengths produced lower classification accuracy, and longer ones did not improve the accuracy, which was also observed in previous studies [51,52]. We developed an algorithm to take out the artefacts from the PPG, GSR, and temperature sensor data. In this algorithm, thresholds were applied to the magnitude of the accelerometer and heart rate to identify artefacts. We considered the following to apply the threshold:

- If the mean accelerometer magnitude for a window was greater than 0.5 m/s${}^{2}$, we discarded all the other signal windows for the same time index. A higher magnitude in the accelerometer signal indicated movement in hand, which induces artefacts in the other signals present in the Shimmer3 GSR+ device.
- A threshold was applied to check if there were more than two unique heart rates across a 2 min window throughout the complete signal. If there were no unique heart rates, then the complete window was discarded and was not used for the study.
- Further, to include the remaining windows, we checked if the heart rate was greater than 35 per window, as the resting state heart rate ranges from 40 to 109 beats per minute [53].

**Feature Extraction and Selection:** We extracted 125 features from the GSR, PPG, and temperature sensor signal windows that were free of artefacts. From the filtered PPG signal, we extracted 46 features as described in the work of Xiao et al. [51]. In total, 4 PPG features were derived from Neurokit2 in the frequency domain, and another 11 PPG features were derived from heart rate. We derived the mean and standard deviation within a window from temperature sensor data. We extracted 43 features from GSR in the time domain using the EDA explorer [54], and 19 features were derived from the tonic, phasic, and SMNA.

In this study, we implemented a hybrid feature selection method. First, we implemented the filtering method to reduce the features by analysing the features for high variance using Analysis of Variance (ANOVA) based on the F1-score. We used the Scikit-Learn Python package with SelectKBest features to implement the ANOVA. Then we used the wrapper method Recursive Feature Elimination with Cross-Validation (RFECV) with RF to reduce the features further. These combinations were used because time and computational complexity can be reduced by excluding redundant features, and model accuracy can be improved [55,56].

**Model Training:** We normalised the selected features before using them to train and test the RF, SVM, and Gaussian Naive Bayes (GNB) classifiers. These models were chosen based on previous work [14,15,30]. We implemented a nested cross-validation hyperparameter optimisation to train these models with a grid search method. The hyperparameters used for RF, SVM, and GNB are listed in Table 1. We performed this optimisation to tune the models and avoid overfitting. In this optimisation, the data from the twelve subjects were split into training and test sets. The data from one subject were used as test data, and the remaining data from the eleven subjects were used for training (LOSO). Using the data from the eleven subjects, we perform 11-fold cross-validation. In each cross-validation, one subject was the validation dataset, and the remaining ten subjects were the training dataset. We perform the hyperparameter optimisation using grid search to configure the model for the ten training datasets. The best model from the grid search cross-validation is evaluated with the last fold. This method is repeated eleven times, and the final cross-validation value is calculated from the mean of all eleven values. Therefore, each of the twelve subjects was tested with a different model because the hyperparameters were different for each model.

**Data Aggregation:** To explore the impact of cognitive load on food choice, we analysed the cognitive load classification results, food choices during both sessions, and the negative affect (from PANAS) experienced during the HL session as it impacted the subjects significantly. The collected data were aggregated and subjected to hierarchical clustering using agglomerative clustering techniques [57,58].

Before conducting the clustering analysis on the food consumption data measured in grams and millilitres, we normalised the values to a scale of 0 to 1 to account for variations in measurement units. This step ensured that the clustering results were not biased by differences in the scales of different food items. We excluded calorie consumption from the analysis due to the inclusion of water, which has zero calories. To facilitate the analysis, we converted the cognitive load classification accuracy results into binary labels. Accuracy above 80% were labelled as 1, indicating high accuracy, while accuracy below 80% were labelled as 0, representing low accuracy. Similarly, participants who reported higher negative affect during the load period compared to before load and after eating were labelled as 1, while the remaining participants were labelled as 0. This labelling approach allowed us to examine the connections between cognitive load, food choice, and negative affect.

## 4. Results

To investigate the relationship between cognitive load and eating behaviour, we conducted a laboratory study where we collected physiological signals for approximately 30 h, NASA-TLX score, PANAS data, and information on the amount of each food consumed. We performed statistical tests on NASA-TLX scores to distinguish between HL and LL task, using it as the ground truth. Additionally, we analysed the food consumption data to understand the subjects’ calorie intake and food choices. We also examined the PANAS data to explore the potential impact of affective states on cognitive load and food choices. Furthermore, we evaluated the accuracy of classifying cognitive load based on the collected physiological signal data. Finally, we aggregated all the data to analyse the relationship between food consumed, cognitive load, and affective states.

### 4.1. Statistical Analysis of NASA-TLX

First, we calculated the mean and standard deviation of the NASA-TLX scores for each of the three HL tasks and LL task for all subjects, shown in Table 2. We observed the largest difference in mean from Table 2 between the Reading Span task (HL) and the Bejeweled 2 Game task (LL). To find the significant difference between the tasks, we first checked the distribution of NASA-TLX scores for each task using the Shapiro–Wilk test. Because the *p*-value of the Shapiro–Wilk test for NASA-TLX was greater than 0.05 for each task, this indicated a normal distribution. Therefore, we performed paired *t*-tests to determine the significant difference between tasks, shown in Table 2. In Table 2, the first three *p*-values were calculated between one of the HL tasks (i.e., Reading Span, Stroop, or N-back tasks) and the LL task (Bejeweled 2 Game task). We found a significant difference between the HL and LL tasks, as the *p*-value is less than 0.05. In Table 2, the last three *p*-values were between the HL tasks. However, there is no significant difference within the HL tasks because the *p*-value is greater than 0.05. This confirms that all the HL tasks were comparable to each other in terms of cognitive load demand.

### 4.2. Statistical Analysis of PANAS

In the present study, we analysed the PANAS questionnaire to assess changes in affective states before and after the cognitive load tasks and after eating in both the sessions. We calculated the mean and standard deviation for positive and negative affective states scores (see Table 3). We then performed the Shapiro–Wilk test to check for data distribution. As the data did not have a normal distribution, we used the Friedman test for statistical analysis. Our *p*-value results are present in Table 3. It can be observed in Table 3 that during the HL session, the mean score for negative affective states before load and after eating was lower than the mean score after load. At the same time, the mean score for positive affective states before load and after eating was higher than the mean score after load in the HL session. However, only the negative affective states showed a significant difference between before load, after load, and after eating (*p*-value = 0.0005) during the HL session. In contrast, the positive affective states showed no significant difference (*p*-value = 0.7165).

To further investigate the significant difference in negative affective states in the HL session, we performed a post-hoc Friedman test. This test revealed a significant difference in negative affective states between after load and after eating (*p*-value = 0.002). However, there was no significant difference in affective states between before and after load combinations or before load and after eating combinations (both had a *p*-value of 0.06). Taken together, our findings suggest that the cognitive load tasks significantly impacted negative affect but not positive affect and that eating after the task helped reduce negative affective states in some individuals during HL sessions. Table 4 presents negative affect scores that were calculated for the PANAS questionnaires answered before the load task, after the load task, and after eating during the HL session. It can be observed from Table 4 that eight subjects had higher negative affect after load when compared to before load and after eating during HL session.

### 4.3. Statistical Analysis of Food Consumed

We analysed the calories consumed of each food for the eight subjects who had higher negative affect before eating. Table 5 shows the significant difference in food consumption between the HL and LL sessions, along with their mean, standard deviation, and direction of change. The direction of change indicates the quantity changes between a HL and a LL session. If the value is above one, there is an increase in the value from LL to HL. If the value is less than one, there is a decrease in the value between LL and HL. Table 5 shows that the *p*-value for chips and grapes is significant between HL and LL sessions. In Table 5, it can also be seen in the direction of change that the consumption of chips and grapes is 1.3 and 1.5 times higher, respectively, at HL session. Chips are considered a salty high-fat food, while grapes are considered healthy sweet food [59]. The increased consumption of these items during the HL session may be attributed to cognitive depletion, which can lead to reduced self-control and heightened susceptibility to foods that are high in sugar and calories.

### 4.4. Feature Selection and Classification Results

Regarding the physiological signals, we discarded some windows in the preprocessing phase of artefact removal based on the criteria mentioned in Section 3.5. About 3.9% and 1.8% of the windows were discarded from the HL and LL tasks, respectively.

**Figure 6:**
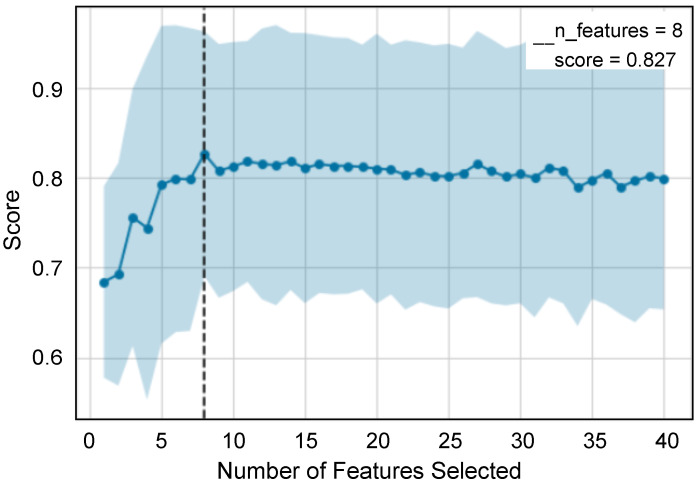
Recursive feature elimination with 12-fold cross validation to select features.

From the artefact-free windows, we extracted 125 features in the time and frequency domains from the GSR, PPG, and the temperature sensor signals. To solve the overfitting problem, we reduced the features. Two types of feature selection methods were used to reduce the features. First, the SelectKBest method was used, in which the features were reduced to the 40 most important ones. Then, the RFECV method with a RF classifier was used. Figure 6 shows the result of the RFECV method, where the x-axis represents the number of features and the y-axis represents the F1-score obtained for these features for the RF model. From Figure 6, it can be seen that the RF model with eight features obtained the best F1-score of 0.827. Table 6 shows the eight features, five of which are from GSR.

**Figure 7:**
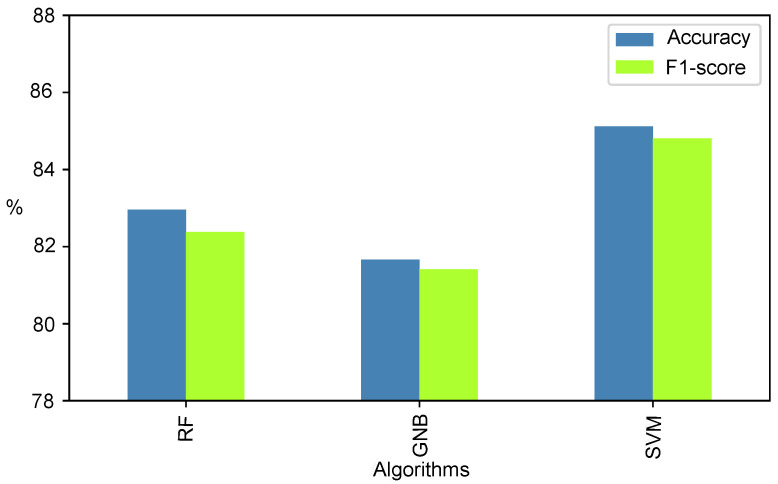
Mean leave-one-subject-out performance of machine learning algorithms for classification of low and high cognitive load. Abbreviation: RF—Random Forest, GNB—Gaussian Naive Bayes, SVM—Support Vector Machine.

We used the eight selected features to train RF, SVM, and GNB models with nested cross-validation for hyperparameter optimisation using a grid search method. Separate models were trained for each subject. The mean accuracy and F1-score for HL and LL task classification for all subjects in the three models are shown in Figure 7. It can be observed that SVM with linear kernel had the accuracy and F1-score for the binary classification greater than 84%. Since SVM had the highest accuracy and F1-score, Table 7 presents the true positive, true negative, false positive, true negative, precision, recall, and F1-score along with the accuracy for each subject in both load sessions. All subjects had accuracy greater than 70%. The hyperparameters used to achieve this accuracy were for the linear kernel with a gamma value of 1, and the C value was mainly 10 and 100.

### 4.5. Relationship between Cognitive Load, Affective States, and Food Choices

**Figure 8:**
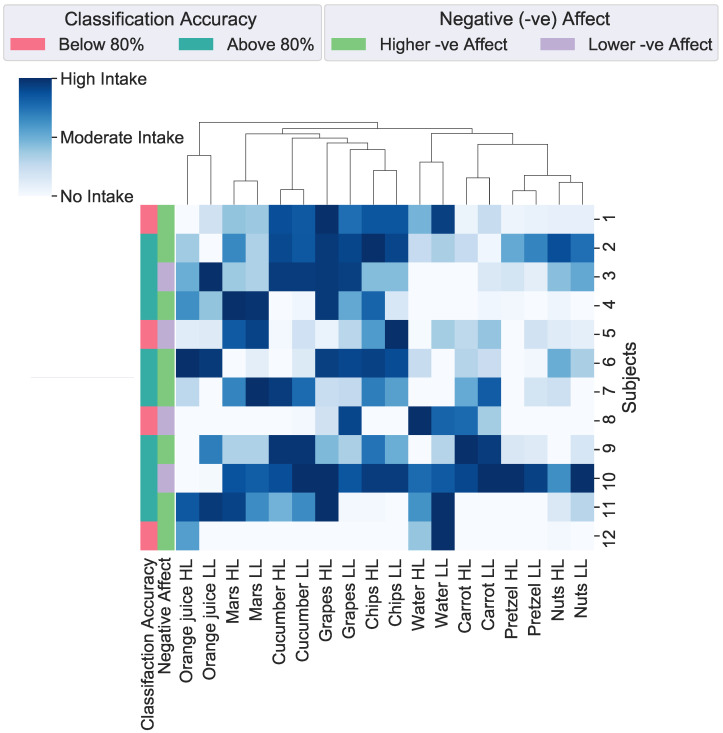
Clustering of aggregate data based on food choices. The aggregated data on the ‘x-axis’ contains food choices made during high- and low-cognitive-load sessions, cognitive load classification accuracy, and the subject’s negative affect during the high-cognitive-load session. The ‘y-axis’ contains the subject number. Abbreviation: HL—High cognitive Load, LL—Low cognitive Load.

Figure 8 illustrates a clustering and heat map displaying the outcomes of the study examining the interplay between cognitive load, negative affect, and food choices. The food choices were categorised into three clusters based on their similarities. The first cluster contained nuts, pretzels, carrots, and water. This food exhibited the lowest consumption among participants in both sessions. Among these choices, only pretzels were considered unhealthy due to their processed nature. The second cluster comprised chips, cucumber, chocolate, and grapes. The foods in the second cluster were the most frequently consumed in both sessions. Notably, among participants experiencing negative affect, there was a significant increase in the consumption of chips and grapes following HL tasks. The third cluster consisted of orange juice, which was moderately consumed compared to the other food choice clusters.

In Figure 8, the labels used to group subjects include “Classification Accuracy” and “Negative (-ve) Affect”. “Classification Accuracy” represents the binary-class cognitive load classification output performed using a SVM with the shimmer GSR+ sensor data. Based on the accuracy scores, the labels are divided into two categories: the first includes classification results “Above 80%”, and the second comprises results “Below 80%”. The “Negative (-ve) Affect” labels are derived from scores obtained through the PANAS during the HL session, as significant differences were observed only in negative affect during this session. Participants who had higher negative affect after the load than before the load and after eating are categorised as having “Higher -ve Affect”, while the remaining participants are categorised as having “Lower -ve Affect”. Within the “Higher -ve Affect” category, there are eight subjects.

Among the eight subjects with “Higher -ve Affect”, approximately 75% achieved accuracy, precision, F1-score, and recall values of 80% or higher. Interestingly, this subset of subjects also exhibited a greater inclination toward consuming unhealthy food choices, particularly chips, during HL sessions. These findings suggest that negative affect and cognitive load can influence food choices, indicating that individuals experiencing negative emotions during HL may have a higher tendency to opt for unhealthy food options. These results have implications for developing interventions to promote healthier food choices and mitigate the adverse effects of cognitive load on dietary habits.

## 5. Discussion

Here, we outline and discuss our contributions with an initial approach to building an eating behaviour assistant system. Further, we address limitations and future work.

### 5.1. Contribution

Our study demonstrates a framework for collecting data to objectively classify cognitive load and its effects on eating behaviour. In this study, we implemented a sequence of tasks, stimuli, and instructions through experimentation software (using PsychoPy, a cross-platform Python package). This implementation has three advantages:

- Other researchers can use this software to replicate our study via the GitHub repository https://github.com/AK1817/Cognitive_load_Experiment.git (accessed on 1 June 2023).
- By using such a software, subjects were not interrupted during the study because we had already provided all instructions on the platform.
- We obtained all timestamps to label our data as HL or LL.

In addition, we could retrieve the questionnaires’ responses through the software we used to create our study.

Our observational analysis of the NASA-TLX and PANAS questionnaires shows that most subjects experienced slightly stronger negative affect immediately after the HL tasks. It is known from previous studies that HL is associated with high or low pleasure in performing a task [60]. One explanation could be that the subjects had the N-back task as the last task in the HL session, and the difficulty level of the dual N-back task could have caused frustration in the subjects. This suggests that the HL could elicit higher negative affect and lower negative affect after eating.

We used hybrid feature selection method combinations to improve accuracy and reduce the time and computational cost of selecting relevant features [55,56]. With filter-based feature selection, we could reduce the time required for feature selection using the wrapper method (RFECV). For the selected features, we found that the GSR features (skin conductance) outnumbered the PPG features. This suggests that the GSR features have a higher weight in the cognitive load classification.

Our study employed a nested cross-validation approach with LOSO to optimise hyperparameters in our classification pipeline. This allowed us to thoroughly evaluate the performance of our model while mitigating overfitting and bias. Each subject was tested on different models that were optimised with specific hyperparameters and trained on the remaining subjects’ data. Hence we could present the individual performance of each subject, providing insights into the model’s accuracy for each participant.

The mean classification accuracy, precision, recall, and F1-score for binary classification were found to be 85.12%, 87.44%, 85.90%, and 84.81%, respectively. These results demonstrate a comparable classification accuracy to previous works [14,15,30]. However, it should be noted that these previous studies either did not present individual subject performance using LOSO or did not employ LOSO cross-validation at all. Moreover, previous studies that achieved classification accuracies greater than 80% utilised more features, ranging from 14 to 42 [14,15]. In contrast, our study achieved an accuracy of 85.12% using only eight features. The lesser number of features is known to simplify the model reducing the number of parameters, decreasing the training time, enhancing generalisation, and avoiding the curse of dimensionality. However, direct comparisons with these studies are limited due to differences in study design and sensor types employed.

The findings of this study shed light on an intriguing relationship between negative affect, cognitive load, and food choices. Notably, among the subjects experiencing higher negative affect, a significant majority exhibited exceptional performance in accuracy, precision, F1-score, and recall, surpassing the 80% threshold. However, a notable observation emerged regarding their food choices during high-cognitive-load sessions, with a distinct preference for unhealthy options, particularly chips. These findings indicate that negative emotions and cognitive load may influence individuals to choose less-healthy food. The implications of these results are noteworthy, suggesting the need for interventions that promote healthier food choices and address the potential negative consequences of cognitive load on dietary habits. Future research should uncover the underlying mechanisms and develop effective strategies to support individuals in making healthier food choices, particularly during periods of cognitive load and negative affect.

### 5.2. Limitations and Future Work

Our study found that half of the subjects consumed high calories during HL sessions, while the other half consumed high calories during LL sessions. However, it is important to note that our study does not aim to establish a direct causal relationship between cognitive load and calorie consumption. We acknowledge that the limited sample size of only 12 subjects is a limitation of our study. Studies in cognitive student engagement assessment [61] and emotion classification [62] have also faced similar challenges with small sample sizes. Furthermore, it is important to consider the findings of previous studies in nutrition science and psychology, which have demonstrated that cognitive load can indeed impact calorie consumption [19,20,35,36]. While our study contributes a valuable framework for objectively measuring cognitive load over an extended period, its generalisability is limited due to the small sample size. Therefore, future studies with larger sample sizes are needed to validate and extend our findings.

The generalisability of our findings to real-life scenarios is limited in our experimental study. Therefore, further validation of the developed models and selected features in real-life contexts is necessary. To conduct such studies, collecting psychological signals and gathering contextual information, including the subject’s location and social environment, is crucial. Additionally, ecological momentary assessments , where subjective questionnaires are answered in real-time using smartphones, can provide valuable insights into the subject’s immediate situation that may not be captured during data collection. These assessments can serve as labels to analyse and interpret the collected data [63].

A limitation of our study is the presence of variations in completion time for the HL tasks among subjects. We observed differences in the number of windows displayed in the HL tasks across subjects, while the number of windows in the LL tasks remained consistent. These discrepancies are evident in Table 7, where the sums of true positive and false negative outcomes for HL tasks, as well as true negative and false positive outcomes for LL tasks, are displayed. These variations can be attributed to task instruction reading time variances and the absence of time constraints in the Reading Span task and the initial round of Stroop tasks. As a result, subjects completed the HL task at different speeds. We identified a significant correlation (Pearson correlation test, *p*-value = 0.017) between the completion time of HL tasks and the occurrence of false negatives. Also, linear regression analysis indicates that as subjects took longer to complete the HL task, their error rate increased, thus resulting in a higher frequency of false negatives (misclassifying HL as LL). However, we did not find a significant correlation (*p*-value = 0.58) between false positives in LL tasks and completion time. Future studies should consider incorporating time pressure as a variable of interest to improve classification accuracy. Importantly, it is worth noting that these variations in completion time did not influence eating behaviour.

## 6. Conclusions

In conclusion, our study aimed to develop an eating behaviour assistance system by conducting experiments in a controlled laboratory environment. To replicate real-life stressors, we implemented experimentation software to ensure uninterrupted data collection, minimising experimenter bias. Through this approach, we obtained approximately 30 h of physiological data available to interested researchers upon request. Our analysis demonstrated the feasibility of objectively classifying cognitive load with an average accuracy of 85.12%. Furthermore, we observed that 75% of subjects experiencing higher negative affect consumed specific foods more frequently when facing HL.

However, it is essential to acknowledge the limitation of our study’s small sample size, which restricts the generalisability of our findings. Therefore, further validation of the developed models and selected features in real-life scenarios is necessary to ensure their practical application. Addressing these limitation and validating the method in real-life settings will strengthen its effectiveness and contribute to its successful implementation. Future research should focus on expanding the sample size and exploring practical strategies for integrating the system into everyday life.

Our research highlights the potential of an eating behaviour assistance system to effectively promote healthier eating habits in individuals experiencing HL in real-time. When taken to a product-level stage, such a system could provide personalised feedback and recommendations based on real-time data. For example, it could suggest healthier recipes that cater to individuals’ cravings, such as baking vegetables with minimal or no oil. Additionally, it could offer alternative palatable food options with lower calorie content, like suggesting popcorn instead of chips, as popcorn has relatively low calories when compared to chips [64]. The system could also provide information on nearby local grocery stores or restaurants where individuals could find these healthier options. Moreover, the system could help individuals resist unhealthy food temptations through mindfulness exercises, such as focusing on breathing or meditation during times of temptation for unhealthy food, especially when experiencing high cognitive load. Similarly, when detecting prolonged periods of HL, the system could integrate spiritual guidance to help individuals access the potential resources of spirituality in their treatment and recovery from eating disorders [65].

## Figures and Tables

**Table 1:** Hyperparamters used for tuning Random Forest (RF), Support Vector Machine (SVM), and Gaussian Naive Bayes (GNB) classifiers.

Model	Parameter	Values
RF	Bootstrap	True, False
	Maximum depth	5, 15, 26, 36, 47, 57, 68, 78, 89, 99, 110
	Maximum features	‘auto’, ‘sqrt’
	Minimum split	2, 5, 10, 15, 20, 25
	Minimum leaf	1, 2, 4, 6, 8, 10, 12
	Number of tree	5, 12, 19, 26, 33, 41, 48, 55, 62, 70
SVM	C	0.1, 1, 10, 100
	Gamma	1, 0.1, 0.01, 0.001
	Kernel	Linear
GNB	Variance smoothing	1 to 1 × $10^{-9}$, Step size = 0.099

**Table 2:** Statistical Analysis of NASA-TLX scores for both sessions with mean, standard deviation (Std), and paired *t*-test. Paired *t*-test *p*-values were calculated between the tasks. Abbreviations: HL—High Cognitive Load, LL—Low Cognitive Load.

Tasks	Mean ± Std	*p*-Value
Reading Span (HL),	64.13±15.74	0.0015
Bejeweled 2 (LL)	39.73±15.60	
Stroop (HL),	54.57±16.80	0.0220
Bejeweled (LL)	39.73±15.60	
N-back (HL),	62.60±16.10	0.0005
Bejeweled 2 (LL)	39.73±15.60	
Reading Span (HL),	64.13±15.74	0.1354
Stroop (HL)	54.57±16.80	
Reading Span (HL),	64.13±15.74	0.7572
N-back (HL)	62.60±16.10	
Stroop (HL),	54.57±16.80	0.1484
N-back (HL)	62.60±16.10	

**Table 3:** Statistical Analysis of PANAS scores with mean, standard deviation (Std) and Friedman’s test *p*-value. These values are calculated for high and low cognitive load tasks, between negative and positive affects before load, after load, and after eating combination. Abbreviation: HL—High Cognitive Load, LL—Low Cognitive Load.

Session	Affect Type	Mean ± Std			*p*-Value
		Before Load	After Load	After Eating	
HL	Negative Affect	13.3±3.7	15.3±3.7	11.5±2.1	0.0005
	Positive Affect	29.4±8.7	27.4±7.8	28.9±8.5	0.7165
LL	Negative Affect	13.4±3.6	12.1±2.2	11.6±8.5	0.2142
	Positive Affect	29.9±7.6	27.6±8.3	27.0±7.8	0.8187

**Table 4:** Negative affect scores were calculated for the PANAS questionnaires answered before the load task, after the load task, and after eating during the high cognitive load session. These scores range between 10 and 50. The scores in bold indicate the increase in the negative affective state just after the load, which is higher than the negative affective state seen before load and also after eating. Abbreviations: HL—High Cognitive Load, NA—Negative Affect.

Load Type	PANAS NA	Subjects and Their Scores											
		l	2	3	4	5	6	7	8	9	10	11	12
HL	Before Load	10	12	17	10	10	13	12	21	10	16	18	11
	After Load	**14**	**13**	13	**12**	10	**21**	**18**	19	**15**	16	**21**	**12**
	After Eating	10	12	11	10	10	11	12	17	10	11	14	10

**Table 5:** Mean, standard deviation (Std), *p*-value, and direction of change in the calories and sugar in grams consumed between the food choices made by the subjects during the high and low cognitive load sessions. Abbreviations: HL—High Cognitive Load, LL—Low Cognitive Load.

Food List	Mean ± Std HL	Mean ± Std LL	*p*-Value	Direction of Change
Potato chips	158.9±93.50	120.1±88.90	0.03	1.3
Pretzel	26.30±47.60	37.70±60.40	0.40	0.7
Grapes	38.00±20.50	25.10±18.30	0.03	1.5
Orange juice	97.10±71.50	86.20±84.70	0.73	1.1
Nuts	89.20±110.9	77.20±94.50	0.54	1.2
Cucumber	6.400±5.300	6.500±4.700	0.88	1.0
Carrot	8.900±11.20	9.600±12.30	0.71	0.9
Mars	121.5±90.30	113.8±88.30	0.68	1.1
Sugar	48.30±25.29	42.08±22.63	0.39	1.1
Total calories	546.2±267.5	476.2±229.1	0.17	1.1

**Table 6:** The eight most important features for the classification of the cognitive load tasks.

Sensor	Feature
GSR	Sudomotor nerve activity count
	Mean raw amplitude
	Maximum power of tonic component
	Maximum power of phasic component
	Standard deviation phasic component
PPG	Mean heart rate
	Standard deviation of heart rate
Temperature	Standard deviation

**Table 7:** Support Vector Machine results for binary classification of high- and low-load cognitive tasks. Abbreviation: TP—True Positive, FN—False Negative, FP—False Positive, TN—True Negative, P—Precision, F1—F1-score, R—Recall, A—Accuracy.

Subject Number	TP	FN	FP	TN	P	F1	R	A
1	35.00	22.00	1.000	46.00	82.43	77.63	79.64	77.88
2	61.00	8.000	0.000	48.00	92.86	93.08	94.2	93.16
3	39.00	12.00	6.000	41.00	82.01	81.62	81.85	81.63
4	38.00	8.000	0.000	47.00	92.73	91.32	91.30	91.40
5	31.00	0.000	20.00	27.00	80.39	74.29	78.72	74.36
6	42.00	16.00	2.000	45.00	84.61	82.84	84.08	82.86
7	31.00	1.000	4.000	49.00	93.29	93.84	94.66	94.12
8	47.00	1.000	25.00	22.00	80.46	70.6	72.36	72.63
9	44.00	2.000	3.000	44.00	94.63	94.62	94.63	94.62
10	37.00	7.000	0.000	48.00	93.64	92.28	92.05	92.39
11	31.00	3.000	0.000	47.00	97.00	96.15	95.59	96.30
12	37.00	3.000	23.00	24.00	75.28	69.43	71.78	70.11
Mean					87.44	84.81	85.90	85.12

## Data Availability

Restricted access to data is made available to researchers in the following link: https://zenodo.org/record/7991589 (accessed on 1 June 2023).

## Abbreviations

The following abbreviations are used in this manuscript:

IMU	Inertial Measurement Unit
BMI	Body Mass Index
RF	Random Forest
SVM	Support Vector Machine
GNB	Gaussian Naive Bayes
LOSO	Leave-One-Subject-Out
PPG	Photoplethysmography
GSR	Galvanic Skin Response
PANAS	Positive and Negative Affect Schedule
ECG	Electrocardiogram
EEG	Electroencephalogram
EOG	Electrooculography
NASA-TLX	National Aeronautics and Space Administration Task Load Index
RFECV	Recursive Feature Elimination with Cross-Validation
SMNA	Sudomotor Nerve Activity
CNN	Convolutional Neural Network
LL	Low Cognitive Load
HL	High Cognitive Load
